# Clinical and echocardiographic trends in percutaneous balloon mitral valvuloplasty

**DOI:** 10.1186/s13019-021-01442-w

**Published:** 2021-04-01

**Authors:** Ofir Koren, Asaf Israeli, Ehud Rozner, Nassem Darawshy, Yoav Turgeman

**Affiliations:** 1 Heart Institute, Emek Medical Center, 21 Yitzhak Rabin Boulevard, 18101 Afula, Israel; 2 Bruce Rappaport Faculty of Medicine, Technion Israel Institute of Technology, Haifa, Israel; 3 Department of Anesthesia, Emek Medical Center, Afula, Israel

**Keywords:** Mitral valve stenosis, Rheumatic heart disease, Balloon Valvuloplasty, Echocardiography, Cardiac Catherization

## Abstract

**Background:**

The prevalence of Rheumatic Mitral Stenosis (MS) has significantly changed over the last decades. We intend to examine patient demographics, Echocardiographic characteristics, procedural success rates, and complications throughout 30-years.

**Methods:**

We conducted a single-center descriptive observational study. The study population consists of patients undergone percutaneous balloon mitral valvuloplasty (PBMV) at Emek Medical Center in Israel from January 1990 to May 2019.

**Results:**

Four hundred seventeen patients underwent PBMV during the study period and were eligible for the study. Age did not change significantly over time (*p* = 0.09). The prevalence of Male and patients who were smoking and had multiple comorbidities such as hypertension, dyslipidemia, ischemic heart disease, and chronic kidney disease became increases over time (*p* = 0.02, *p* = 0.02, *p* = 0.001, *p* = 0.01, *p* = 0.02, and *p* = 0.001, respectively). Wilkins score and all its components increased over time, and the total score was higher in females (*p* = 0.01). Seventy-nine (18.9%) patients had complications. The rate of complications did not change over decades. Patients with Wilkins score > 8, post-procedural MR of ≥2, and post-procedural MVA < 1.5 had the highest risk for the need of Mitral valve replacement (MVR) surgery in 2 years following PBMV (3.64, 4.03, 2.44, respectively, CI 95%, *p* < .0001 for all). The median time in these patients was 630 days compared to 4–5 years in the entire population. Patients with Post-procedural MR of ≥2 and post-procedural MVA < 1.5 had ten times risk for developing heart failure (HR 9.07 and 10.06, respectively, CI 95%, *P* < .0001).

**Conclusion:**

Our research reveals trends over time in patients’ characteristics and echocardiographic features. Our study population consists of more male patients with multiple comorbidities and more complex and calcified valvular structures in the last decade. Wilkins score > 8, post-procedural MR of ≥2, and post-procedural MVA < 1.5 cm^2^ were in-depended predictors for the time for surgery and heart failure hospitalization.

## Introduction

Rheumatic heart disease (RHD) is a serious cardiac complication of an immune-mediated infectious disease known as a rheumatic fever caused by *Streptococcus pyogenes* infection in childhood. The most prominent late manifestation of RHD is valvular dysfunction, which primarily affects the mitral and aortic valves. The inflammatory process leads to a significant thickening and calcification of the valves and subvalvular apparatus, resulting in stenotic and insufficient leaflet function [1–3].

The prevalence of rheumatic fever in Israel was estimated in a large cohort study was 0.12%. A downward trend from the early 80s and highly influenced by Ethiopia’s latest immigration and the former Soviet Union. In this cohort, the prevalence of valvular disease was 15.7% in patients who had ARF [4].

Percutaneous balloon mitral valvuloplasty (PBMV) was first introduced in the early 1980s by Kanji Inoue, a Japanese surgeon. He conceived that a narrowed pliable valve could be expanded by splitting the valve commissures using a balloon inflated with high pressure. Before the advent of PBMV, the recommended treatment approach for severe symptomatic narrowing of the mitral valve was surgery (i.e., surgical commissurotomy) [5, 6].

Over the years, PBMV has been extensively studied and demonstrates a high success rate and low incidence of complications than the surgical approach [7–11].

The valve and subvalvular mechanism must be elastic and free of calcifications for PBMV to be feasible; before PBMV, these conditions are evaluated using several echocardiographic indices. The most common and recommended measure is the Wilkins score, which includes four main key criteria: mobility, thickness, and calcification of the mitral leaflets and involvement of the subvalvular mechanism. Each measure is scored from one to four according to its severity. A total score of eight or fewer in a patient with symptomatic mitral stenosis (MS) and nonsignificant mitral insufficiency predicts good results and a low complication rate. A total score greater than eight, especially when the mitral valve insufficiency is more than moderate, predicts a low success rate and a high complication rate compared with the surgical approach [12–15]. Considering this experience, the American Cardiology Association (ACC) and the American Heart Association (AHA) recommended **PBMV** for the treatment of patients with pliable rheumatic MS. [16]

### Rationale of the study

Our institute has performed Percutaneous balloon mitral valvuloplasty (PBMV) for significant mitral valve stenosis for almost three decades. PBMV is the treatment of choice provided that valvular morphology is favorable. The study aimed to examine whether the constant decline in RHD incidence in the western world is also accompanied by clinical and echocardiographic changes, affecting treatment efficacy and outcome.

## Material & Methods

### Planning of the research

**Fig. 1:**
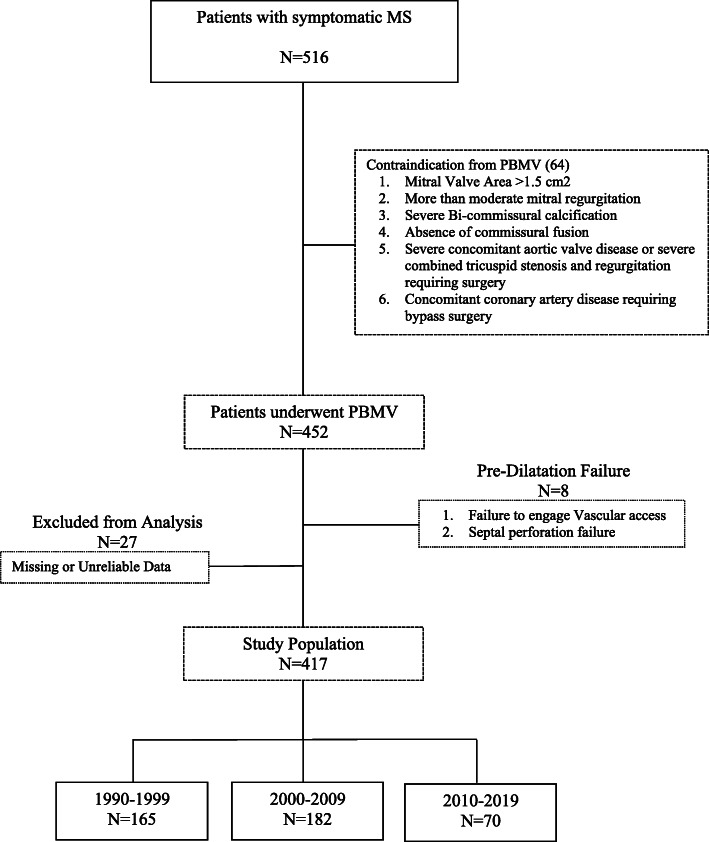
Study design

We designed a retrospective observational descriptive cohort study at our heart institute at Emek Medical Center in Israel. The study population included patients who hospitalized significant symptomatic MS from January 1990 to May 2019. The patient’s medical information was collected from the hospital’s computer systems and Clalit health data service, Orion, Ofek, and Chameleon (Fig. 1).

Patients with mitral valve area > 1.5 cm^2^, moderate or severe mitral insufficiency, evidence of a clot in the left atrium, severe concomitant valvular disease, or patients requiring bypass surgery have been excluded from the study (Table 1. Criteria for inclusion and exclusion from the study. Supplementary). Patients eligible for the study were divided according to three decades: 1990–1999, 2000–2009, and 2010–2019. Patients were followed to evaluate disease progression.

**Table 1:** Patient characteristics during the study period (*N* = 417). Cochran-Armitage test for trend

Patient characteristics	1990–1999 (*N* = 165)	2000–2009 (*N* = 182)	2010–2019 (*N* = 70)	*P*-value (test trend)	Age-adjusted
Age (Mean ± SD years) (Median; range)	57.7 ± 10.8 (57; 28–84)	55.3 ± 11.0 (56; 23–88)	55.1 ± 11.3 (54; 29–80)	.09	–
Sex (female)	139 (84.2)	147 (80.8)	48 (69.6)	**.02**	**.02**
Obesity (BMI > 30)	79 (48.2)	71 (39.2)	29 (41.4)	.19	.27
Smoking	48 (29.8)	68 (37.8)	37 (52.9)	**.001**	**.001**
Hypertension	66 (40.5)	81 (45.3)	38 (54.3)	.06	**.02**
Dyslipidemia	69 (42.1)	80 (45.2)	40 (58.0)	**.04**	**.02**
Diabetes mellitus	44 (27.0)	43 (24.2)	22 (31.1)	.64	.11
Chronic kidney failure	22 (13.6)	26 (14.7)	24 (34.8)	**.001**	**.001**
Ischemic heart disease	45 (27.6)	49 (27.7)	28 (40.6)	.10	**.01**
CABG	7 (4.3)	10 (5.6)	6 (9.0)	.19	.20
Atrial fibrillation	79 (48.2)	92 (50.5)	40 (57.1)	.24	.63
Stroke	21 (13.0)	26 (14.4)	10 (14.7)	.68	.74
Endocarditis	6 (3.8)	3 (1.7)	1 (1.5)	.21	.27

*CABG* coronary artery bypass graft

This study’s main objective was to examine trends over time regarding patient demographics, echocardiographic features of the valves and sub-valvular apparatus, success rates, and complications.

The primary endpoint was the procedural success rate, defined as the mitral valve area’s dilatation (MVA) to 1.5 cm^2^ or more (MVA ± 1.5 cm^2^). The study’s secondary endpoints were a 2-years composite of heart failure hospitalization, functional capacity deuteriation (asses by the New York Heart Association (NYHA) class), and the need for mitral valve surgery.

### Statistics

The trend was performed by linear regression for continuous data, the Cochran-Armitage test for categorical data, and the Jonckheere-Terpstra test for ordinal data (Wilkins subscore). Trends were adjusted for age, Bonferroni pairwise comparisons were performed to assess differences between decades. Graphs of the trend over a year were created using spline interpolation. Receiver operating characteristic curve (ROC) analysis was performed to determine the Wilkins and the MVA cutoff that would best predict complications, time to mitral valve surgery, and heart failure hospitalization. Logistic regression was performed to determine predictors of complications and successful technical operations. These were then adjusted for age. The multivariate prediction model was then performed using the statistically significant age-adjusted univariate predictors.

Statistical significance is considered when *p* < 0.05. All analyses were performed using SPSS (version 23, IBM Inc.).

### Ethics

The Ethics Committee approved the study of the hospital following the Helsinki Convention No. EMC-0076-17. The Ethics committee waived informed consent due to the study’s methodology and patient data confidentiality.

## Results

Four hundred seventeen patients (334 Females; aged 23–88) underwent PBMV over 30-years study period. Mean age of the overall population was 56.2 ± 11.0 years. Analysis by years revealed no significant trend in age (*p* = 0.09) (Table 1). There was a significant increase in male patients’ rates, smoking, dyslipidemia, and chronic kidney disease, and this remained true after adjusting for age (*p* = 0.02, *p* = 0.001, *p* = 0.02, and *p* = 0.001, respectively). after adjusting for an age - there was also an increasing trend in hypertension and ischemic heart disease (*p* = 0.02 and *p* = 0.01).

**Fig. 2:**
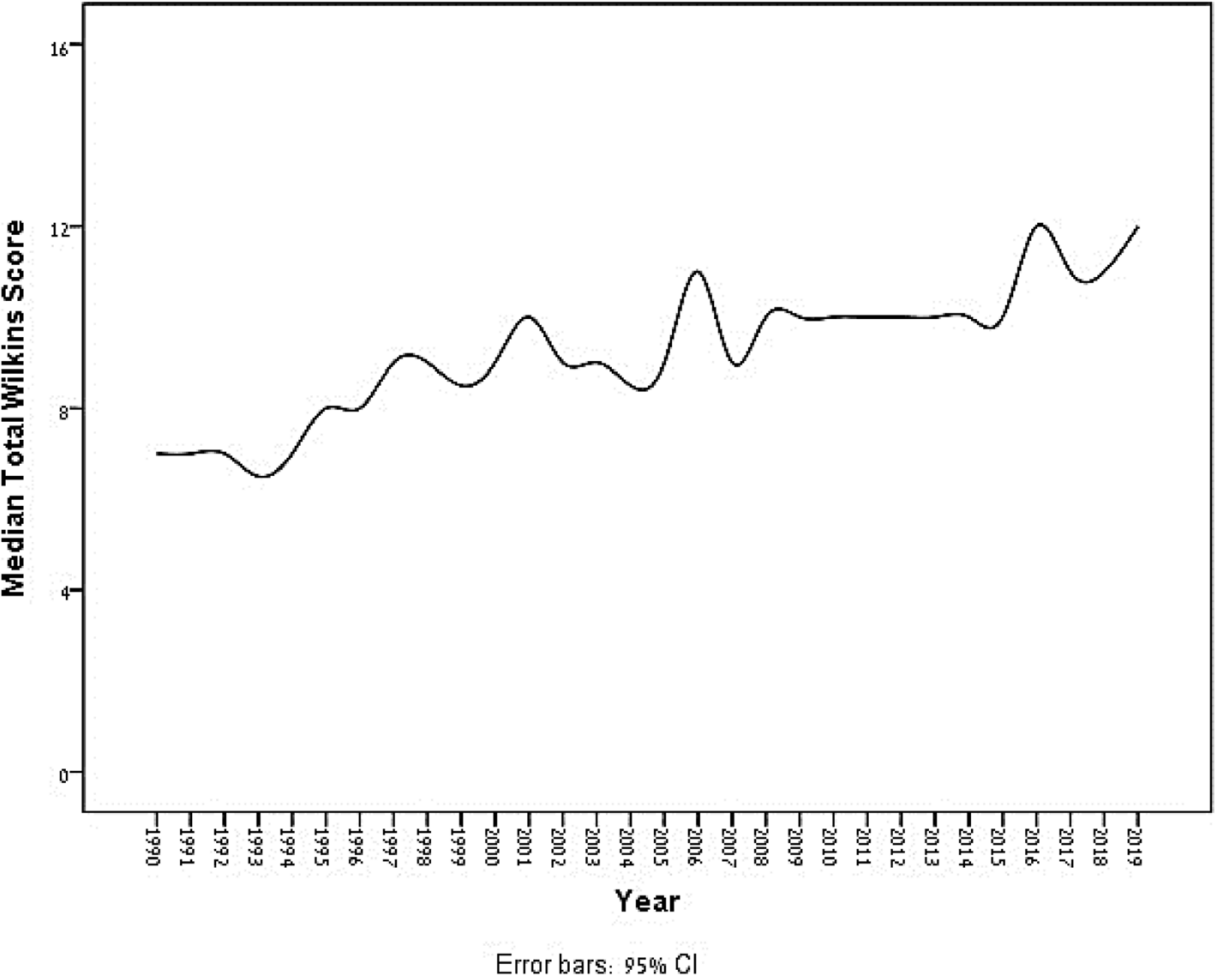
Yearly median Wilkins Score

Linear regression analysis of the mean Wilkins score revealed that the Wilkins score increased by 1.379 per decade and 0.151 per year, and median Wilkins increased on average by 1.295 per decade and 0.145 per year (Fig. 2). The Jonckheere-Terpstra test showed a significant trend for all Wilkins variables (calcification of leaflets, calcification of subvalvular apparatus, mobility, and thickening) (Table 2). Wilkins score was not associated with age (r = 0.003, *p* = 0.95), but it was significantly higher in male patients (*p* < 0.01), with mean Wilkins 9.27 as opposed to 8.77 in females.

**Table 2:** Wilkins score and its components over decades (*N* = 417)

	Decades				Pairwise Bonferroni Corrected *p*-value		
	1990–1999 (1) (***N*** = 166)	2000–2009 (2) (***N*** = 181)	2010–2019 (3) (***N*** = 70)	Jonckheere -Terpstra ***P***-value	1 vs 2	1 vs 3	2 vs 3
Wilkins score, mean ± SD (median, range)	7.7 ± 1.3 (8; 4–12)	9.4 ± 1.3 (9; 5–12)	10.3 ± 1.1 (10; 7–13)	.001^1^	.001	.001	.001
< 8 8	71 (42.8) 95 (57.2)	10 (5.5) 171 (94.5)	1 (1.4) 69 (98.6)	.001	.001	.001	.001
< 9 ≥9	124 (74.7) 42 (25.3)	43 (23.8) 138 (76.2)	4 (5.7) 66 (94.3)	.001	.001	.001	.001
< 10 ≥10	154 (92.8) 12 (7.2)	100 (55.4) 81 (44.6)	14 (20.0) 56 (80.0)	.001	.001	.001	.001
< 11 ≥11	162 (97.6) 4 (2.4)	145 (80.1) 36 (19.9)	39 (55.7) 31 (44.3)	.001	.001	.001	.001
Calcification of leaflets	1.92 ± 0.80 (2; 1–4)	2.26 ± 0.84 (3; 1–4)	2.80 ± 0.75 (3; 1–4)	.001	.001	.001	.001
Calcification of subvalvular apparatus	2.05 ± 0.72 (2; 1–4)	2.27 ± 0.85 (2; 1–4)	2.60 ± 0.73 (3; 1–4)	.001	.02	.001	.009
Mobility	1.90 ± 0.77 (2; 1–4)	2.25 ± 0.64 (2; 1–4)	2.43 ± 0.71 (2; 1–4)	.001	.001	.001	.051
Thickening	1.84 ± 0.81 (2; 1–4)	2.60 ± 0.60 (3; 1–4)	2.49 ± 0.61 (2; 1–4)	.001	.001	.001	.32

^1^Linear regression

Data is mean ± SD (Median; range)

**Fig. 3:**
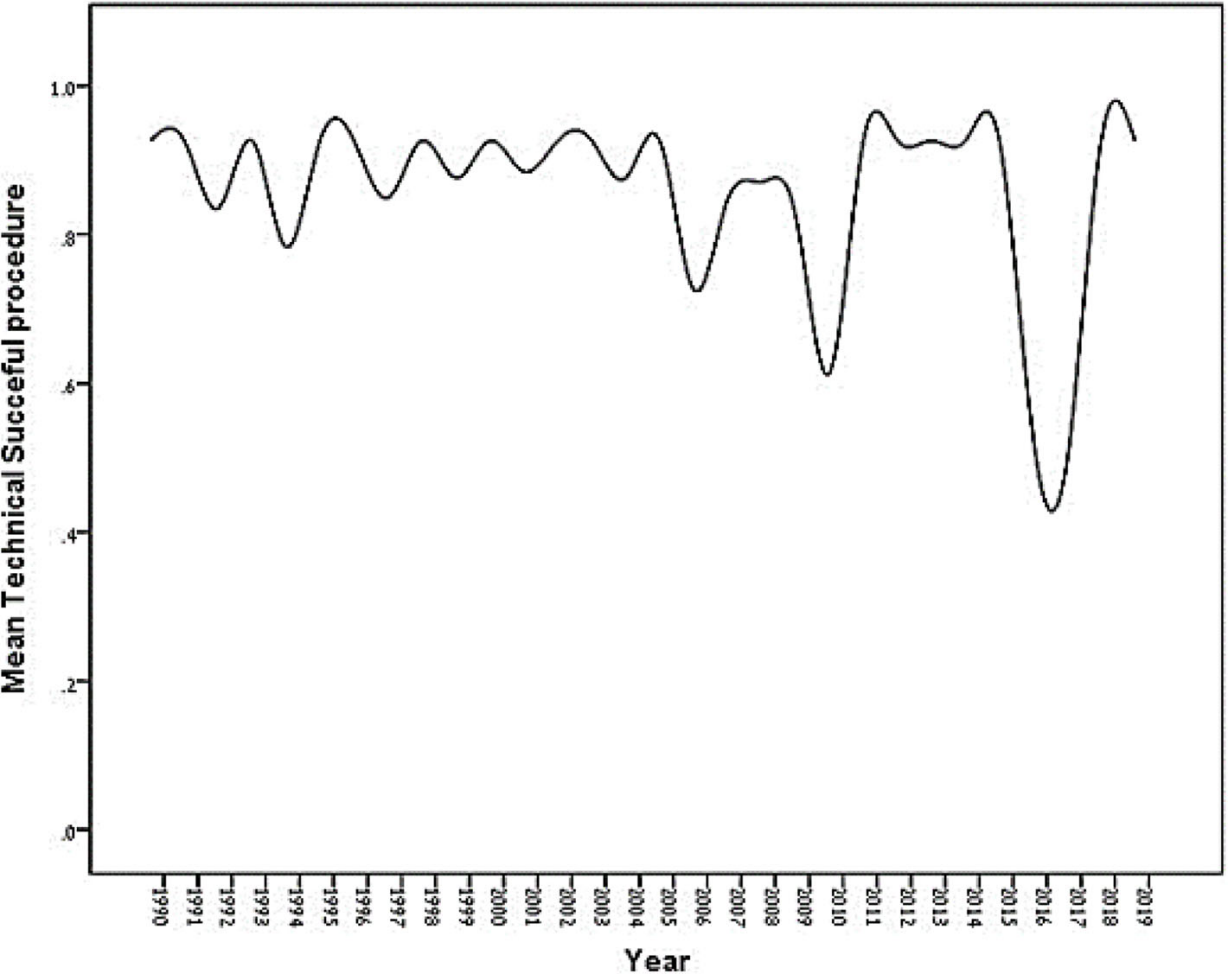
Yearly rate of successful procedures

Out of the 417 patients, 79 (18.9%) had complications. Fifty-six (13.4%) patients exhibit worsening in mitral regurgitations, 9 (2.2%) tamponades, 6 (1.4%) ASD, 1 (0.2%) stroke, and 7 (1.7%) required urgent surgery. The complications rate did not change over decades. Age, hypertension, LVEF, and grade 4 Mobility predicted the occurrence of complications. After correcting for age, hypertension and LVEF were no longer predictive (*p* = 0.07 and *p* = 0.11) (Table 3). Wilkins score of 7.5 was also found to be a cutoff predictor for procedural complications with a 73% sensitivity and 68% specificity (AUC = 0.647 [0.447–0.669], CI 95%, *p* < .003). Hypertension decreased the odds of a technically successful operation after adjusting for age (*p *= 0.02). A Higher Wilkins score predicted a technically successful operation (*p* = 0.04, and *p* = 0.002, respectively). It remained true after adjusting for age (*p* = 0.05, and *p* = 0.004, respectively). The third decade had significantly lower technically successful operations as compared to the first decade (Fig. 3). Multivariate logistic regression analysis revealed that after adjusting for age, decade (*p* = 0.02) and hypertension (*p* = 0.02) were significant predictors of a technically successful operation while Wilkins score as a sole independent factor was not significant (*p* = 0.74, and *p* = 0.22 respectively) (Table 4).

**Table 3:** Patient characteristics and procedural's complications

Patient characteristics	Complications (***N*** = 200)	Nocomplications (***N*** = 217)	***P***-value	Age-adjusted		OR
				OR	***P***-value	
Age (Mean ± SD years) (Median; range)	57.5 ± 10.2 (57; 29–84)	55.0 ± 11.7 (55; 23–88)	**.09**	1.02	–	–
Sex			.12		.09	
Female	166 (49.7)	168 (50.3)		1.47		1.54
Male	33 (40.2)	49 (59.8)		1.00		1.00
Obesity (BMI > 30)	91 (45.7)	88 (40.7)	.31	1.23	.48	1.15
Smoking	70 (35.2)	83 (39.2)	.40	0.84	.32	0.82
Hypertension	100 (50.8)	85 (39.5)	**.02**	1.58	.07	1.46
Dyslipidemia	95 (48.5)	94 (43.9)	.36	1.20	.56	1.12
Diabetes mellitus	55 (28.1)	54 (25.2)	.52	1.16	.82	1.05
Chronic kidney failure	35 (17.9)	37 (17.5)	.92	1.03	.95	0.98
Ischemic heart disease	56 (28.9)	66 (30.7)	.69	0.92	.20	0.74
CABG	8 (4.1)	15 (7.0)	.21	0.57	.20	0.56
Atrial fibrillation	101 (50.5)	110 (50.9)	.93	0.98	.49	0.87
Stroke	28 (14.3)	29 (13.6)	.83	1.06	.95	1.02
Endocarditis	4 (2.1)	6 (2.9)	.61	0.72	.60	0.71
Decade			.83		.09	
1990–1999	73 (44.2)	92 (55.8)		1.00		1.00
2000–2009	98 (53.8)	84 (46.2)	.07	1.47	.06	1.51
2010–2019	29 (41.1)	41 (58.9)	.69	0.89	.75	0.91
Wilkins score, mean ± SD (median, range)	9.0 ± 1.5 (9; 5–12)	8.8 ± 1.7 (9; 4–13)	.12	1.10	.14	1.10
Wilkins by score	Rate		.37			
< 8	35 (42.7)	47 (57.3)		1.00		1.00
≥ 8	37 (42.0)	51 (58.0)	.31	1.29	.29	1.02
≥ 9	53 (54.1)	45 (45.9)	.08	1.42	10	1.40
10	41 (52.6)	37 (47.4)	.54	1.13	.52	1.14
11+	34 (47.9)	37 (52.1)	.34	1.25	.34	1.25
Calcification of leaflets			.72		.76	
1	41 (47.7)	45 (52.3)	–	1.00	–	1.00
2	89 (47.1)	100 (52.9)	.92	0.98	.89	0.97
3	56 (51.9)	52 (48.1)	.62	1.16	.66	1.14
4	14 (41.2)	20 (58.8)	.49	0.75	.50	0.76
Calcification of subvalvular apparatus			.80		.79	
1	33 (45.8)	39 (54.2)	–	1.00	–	1.00
2	93 (47.0)	105 (53.0	.84	1.06	.72	1.11
3	63 (51.2)	60 (48.8)	.40	1.28	.34	1.33
4	11 (45.8)	13 (54.2)	>.99	1.00	.86	1.09
Leaflets Mobility			**.06**		.11	
1	28 (39.4)	43 (60.6)	–	1.00	–	1.00
2	117 (50.6)	114 (49.4)	.14	1.50	.18	1.46
3	44 (44.4)	56 (55.6)	.62	1.17	.79	1.09
4	11 (73.3)	4 (26.7)	.**03**	4.03	.**04**	3.83
Leaflets Thickening			.28		0.30	
1	27 (39.7)	41 (60.3)	–	1.00	–	1.00
2	85 (47.0)	96 (53.0)	.31	1.34	.29	1.37
3	81 (53.6)	70 (46.4)	.07	1.71	.07	1.73
4	7 (41.2)	10 (58.8)	.95	1.04	.78	1.16
LVEF	58.0 ± 4.7 (60; 45–83)	58.9 ± 4.2 (60; 40–72)	**.03**		0.11	0.96

*LVEF* left ventricular ejection fraction

**Table 4:** Patient characteristics and technically successful procedure

Patient characteristics	Successful (***N*** = 389)	Not successful (***N*** = 27)	***P***-value	Age-adjusted		
				OR	***P***-value	OR
Age (Mean ± SD years) (Median; range)	56.4 ± 11.0 (56; 23–88)	52.8 ± 10.0 (52; 36–76)	.10	1.03	–	–
Sex			.40		.38	
Female	313 (94.0)	20 (6.0)		1.46		
Male	75 (91.5)	7 (8.5)		1.00		
Obesity (BMI > 30)			.88		.70	
Yes	166 (93.3)	12 (6.7)		0.94		
No	221 (93.6)	15 (6.4)		1.00		
Smoking	144 (94.1)	9 (5.9)	.77	1.13	.85	
Hypertension			**.052**		**.02**	
Yes	168 (90.8)	17 (7.2)		0.46		0.38
No	221 (95.6)	10 (4.4)		1.00		1.00
Dyslipidemia	177 (93.7)	12 (6.3)	.85	1.08	.93	
Diabetes mellitus	100 (92.6)	8 (7.4)	.60	0.80	.41	
Chronic kidney failure	67 (93.1)	5 (6.9)	.91	0.94	.85	
Ischemic heart disease	111 (91.0)	11 (9.0)	.20	0.60	**.05**	
CABG	21 (91.3)	2 (8.7)	.69	0.74	.69	
Atrial fibrillation	194 (91.9)	17 (8.1)	.19	0.59	.10	
Stroke	52 (92.9)	4 (7.1)	.86	0.91	.79	
Endocarditis	9 (90.0)	1 (10.0)	.67	0.64	.67	
Decade			**.01**		**.02**	
1990–1999	159 (97.0)	5 (3.0)		1.00		1.00
2000–2009	170 (93.4)	12 (6.6)	.32	0.45	.18	0.48
2010–2019	60 (85.7)	10 (14.3)	**.007**	0.19	**.005**	0.20
Wilkins score, mean ± SD (median, range)	8.8 ± 1.5 (9; 5–13)	10.0 ± 2.4 (9; 4–12)	**.001**	0.60	**.001**	0.61
Category	Rate					
< 8	76 (93.8)	5 (6.2)	.90	1.00	.89	1.00
≥8	313 (93.4)	22 (6.6)		0.94		0.93
< 9	163 (96.4)	6 (3.6)	**.04**	1.00	**.05**	1.00
≥ 9	226 (91.5)	21 (8.5)		0.40		0.39
< 10	257 (96.3)	10 (3.7)	**.002**	1.00	**.004**	1.00
≥ 10	132 (88.6)	17 (11.4)		0.30		0.30
Calcification of leaflets			.25		.28	
1	82 (95.3)	4 (4.7)	–	1.00	–	1.00
2	176 (93.6)	12 (6.4)	.57	0.72	.54	0.70
3	102 (94.4)	6 (5.6)	.79	0.83	.75	0.81
4	29 (85.3)	5 (14.7)	.08	0.28	.08	0.29
Calcification of subvalvular apparatus			**.001**		**.001**	
1	68 (95.8)	3 (4.2)	–	1.00	–	1.00
2	189 (95.5)	9 (4.5)	.90	0.93	.96	0.97
3	117 (95.1)	6 (4.9)	.82	0.86	.85	0.87
4	15 (62.5)	9 (37.5)	.**001**	0.07	.**001**	0.08
Leaflets Mobility			.55		.48	
1	68 (95.8)	3 (4.2)	–	1.00	–	1.00
2	216 (93.9)	14 (6.1)	.59	0.68	.52	0.66
3	92 (92.0)	8 (8.0)	.34	0.51	.26	0.47
4	13 (86.7)	2 (13.3)	.20	0.29	.17	0.27
Leaflets Thickening			.30		.40	
1	65 (95.6)	3 (4.4)	–	1.00	–	1.00
2	168 (93.3)	12 (6.7)	.51	0.65	.53	0.66
3	142 (94.0)	9 (6.0)	.66	0.73	.65	0.74
4	14 (82.4)	3 (17.6)	.08	0.22	.11	0.25
ECHO characteristics						
LVEF	58.5 ± 4.5 (60; 50–60)	58.2 ± 3.7 (60; 50–60)	.79	1.01	.71	1.02
Pressure gradients over MV	14.73 ± 6.24 (14; 3–60)	12.74 ± 7.29 (12; 5–36)	.02	1.06	.06	1.09
MVA	1.59 ± 0.25 (1.6; 0.7–2.5)	1.38 ± 0.05 (1.4; 1.3–1.5)	.001	115.5	.001	109.4

*LVEF* left ventricular ejection fraction, *MVA* Mitral valve area

Eighty-two patients (19.7%) had mitral valve surgery (MVR) during the study period. Patients with Wilkins score > 8, post-procedural MR of ≥2, and post-procedural MVA < 1.5 had the highest hazard ratio (HR), predicting the need for Mitral valve replacement (MVR) surgery in 2 years following PBMV (3.64, 4.03, 2.44, respectively, CI 95%, *p* < .0001 for all) (Table 5). ROC curve analysis revealed cutoff points of Wilkins score of 7.5 and post-procedural MVA < 1.35 cm^2^ to best predict a 2-years MVR [80.8, 80.5%, AUC 0.62 [0.552–0.687], CI 95% *p* = 0.002, and 80.3, 76.3%, AUC 0.664 [0.407–0.782], *p* < .0001, cutoff points for sensitivity and specificity, respectively).

**Table 5:** The two-years Hazard ratio for the need of mitral valve surgery, Heart failure hospitalization events, and composite endpoints ^a^ among the study population

Characteristics [N]	HR for MVR surgery [CI %95%]	HR for HF hospitalization [CI 95]	HR for Composite Endpoints ^a^ [CI %95%]	***P***-value
**Wilkins Score > 8**	3.648 [2.34–5.68]	2.724 [1.76–4.21]	2.330 [1.57–3.45]	<.0001
**Post-Procedural MR ≥ 2**	4.03 [2.05–7.92]	9.07 [4.73–17.38]	3.127 [1.63–5.96]	<.0001
**Post-Procedural MVA < 1.5**	2.44 [1.63–3.64]	10.06 [6.89–14.51]	2.33 [1.62–3.35]	<.0001

*HR* Hazard ratio, *HF* heart failure

^a^ 2-years composite endpoints for Heart Failure hospitalization, Mitral Valve Surgery, and worsening functional capacity (assessed by the New York Heart Association classification)

Patients with Post-procedural MR of ≥2 and post-procedural MVA < 1.5 had ten times the risk for developing heart failure (HR 9.07 and 10.06, respectively, CI 95%, *P* < .0001) (Table 5).

The secondary endpoints of a 2-year composite of heart failure hospitalization, functional capacity deuteriation (asses by the New York Heart Association (NYHA) class), and the need for mitral valve surgery were observed in 101 (24.4%) patients. Wilkins score > 8, Post-procedural MR of ≥2, or post-procedural MVA < 1.5 predict risk of two to three times for developing composite endpoints.

**Fig. 4:**
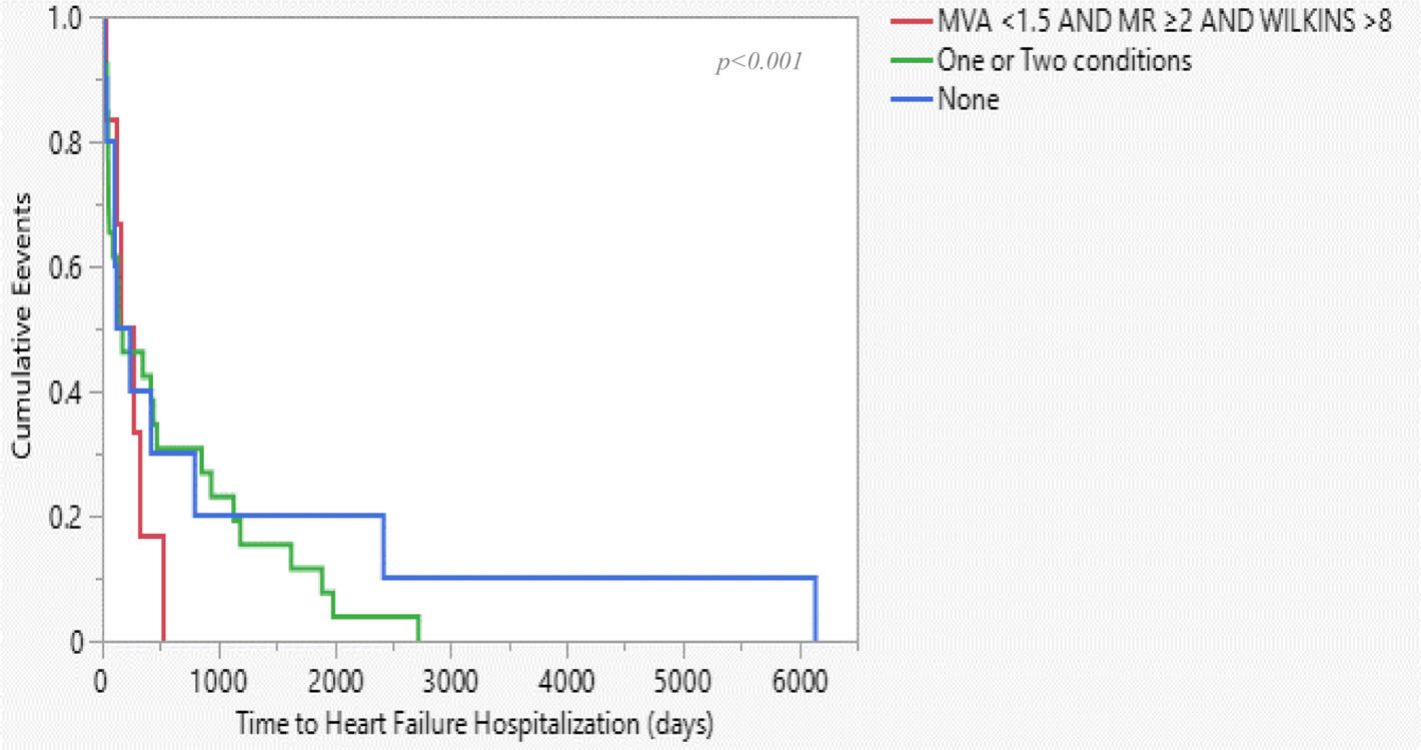
Kaplan-Meier curve of Time to Heart failure hospitalization among three-defined groups

**Fig. 5:**
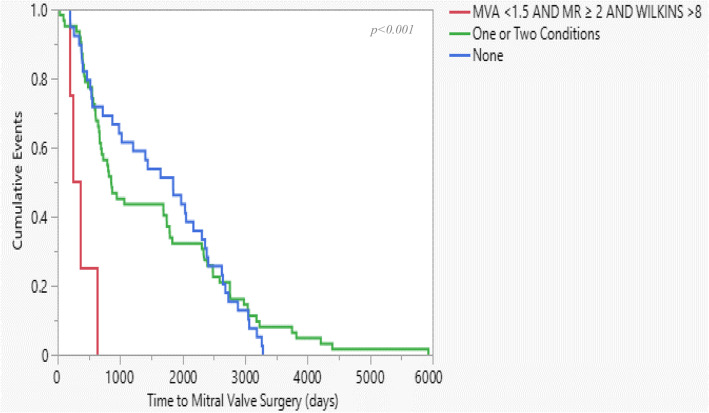
Kaplan-Meier curve of time to Mitral Valve Replacement (MVR) surgery among three-defined groups

We divided the study population into three groups based on three main characteristics and procedural outcomes: Wilkins score > 8, post-procedural MR of ≥2, and post-procedural MVA < 1.5. each group consists of patients who exhibit all three characteristics, one or two or none of the characteristics. Kaplan-Meyer curve reveals that patients who exhibit all three characteristics were hospitalized due to heart failure and undergo MVR surgery significantly earlier than patients who exhibit one or two characters. The median time for MVR was 4 to 5 years for the entire population, while the median time for patients who exhibit all three characteristics was 630 days (*p* < .0001). Patients who did not exhibit any character had the longest HF and MVR time-free events than the other groups (Fig. 4 and Fig. 5, respectively).

## Discussion

Changes and trends in patient characteristics and valve morphology may be an integral part of the declining trend in the incidence of the disease reported in all developed countries throughout the world.

Over time we could see a higher prevalence of male patients and a higher incidence of comorbidities, namely, smoking, hypertension, dyslipidemia, chronic kidney disease, and ischemic heart disease. The valvular and sub-valvular apparatus’s Echocardiographic appearance was also changed with a higher prevalence of severely calcified and thickened leaflets that may be attributed to PBMV procedures becoming more difficult and susceptible for complication.

One explanation for these trends could be derived from the gender shift seen over time. RHD is considered a female-predominant disease with female prevalence exceeding twice the prevalence of males [16]. The gender-shift alongside the higher prevalence of comorbidities may represent a unique and distinct feature of Male associated rheumatic heart disease.

Our growing experience, the meticulous selection of patients, and the development of the PBMV technique could explain the absence of an increase in complication rates over the years —that is, from the use of the balloon-over-the-wire technique to the Inoue-balloon-catheter system, which enables easier maneuvering, gradual balloon expansion, and the absence of a stiff guidewire, leading to a higher incidence of left ventricle rupture.

The long follow-up period allows us to assess the in-depended factors for long-term outcomes measured as time-free from mitral valve surgery and functional deterioration in daily life activities. We identified three major factors that contribute the most for the prediction of MVR surgery and the functional improvement and can help both the patient and the physician in gaining a better understanding of the long-term outcome of PBMV, aid in patients selection, assist the operator in planning a desire post-procedure MVA and proper medical treatment and adequate follow-up following the procedure.

### Limitations of the study

This study included prospective data collection and a retrospective analysis of research that began more than 20 years ago. The data analysis was based on echocardiographic tests in our institute and inserted into the hospital’s computer system. Due to technical limitations, we have limited ability to reevaluate these measurements. Patient medical data regarding hospitalizations and visits to medical centers in our region were loaded on a ‘Clalit’ database several years after initiating the first PBMV procedures. We assume that PBMV or MVR surgery data and were not coordinated with our center were missing. We estimated an uncertainty rate of missing data of no more than 3 %. This rate, in our analysis, does not change the results and conclusions of the study.

## Conclusions

Our study revealed a gender shift seen over time with males becoming more common than in the early 80s, accompanying a higher prevalence of comorbidities, a more complex valvular structure, and less ideal morphology for recommended percutaneous approach urging us for further research in Male-associated RHD. Identifying long-term predictors for procedure outcome and quality of life in our study could contribute to better patient selection and procedural planning.

## Abbreviations

PBMV: Percutaneous balloon mitral valvuloplasty
MVR: Mitral valve replacement
RHD: Rheumatic heart disease
MVA: Mitral valve area
